# Seropositivity and risk factors for *Brucella* in dairy cows in urban and peri-urban small-scale farming in Tajikistan

**DOI:** 10.1007/s11250-013-0534-9

**Published:** 2014-01-12

**Authors:** Elisabeth Lindahl, Nosirjon Sattorov, Sofia Boqvist, Izzatullo Sattori, Ulf Magnusson

**Affiliations:** 1 Division of Reproduction, Department of Clinical Sciences, Swedish University of Agricultural Sciences, P.O. Box 7054, 750 07 Uppsala, Sweden; 2 Tajik Agrarian University, P.O. 734003, Dushanbe, Tajikistan; 3 Division of Food Safety and Bacteriology, Department of Biomedical Sciences and Veterinary Public Health, Swedish University of Agricultural Sciences, P.O. Box 7028, 750 07 Uppsala, Sweden

**Keywords:** Zoonotic disease, Central Asia, *Brucella*, Bovine, Cattle, Risk factor

## Abstract

In this cross-sectional study, we assessed and mapped the seroprevalence of brucellosis in small-scale dairy farming in an urban and peri-urban area of Tajikistan and investigated factors associated with seropositivity. As urban and peri-urban farming is both an opportunity to improve the livelihood for small-scale farmers and a potential public health hazard, studies are warranted to reveal possible peculiarities in the epidemiology of brucellosis in this type of dairy farming. In total, 904 cows of breeding age belonging to 443 herds in 32 villages were serologically tested with indirect enzyme-linked immunosorbent assay (ELISA) and positive samples confirmed with competitive ELISA. Two logistic regression models were used to investigate an association between seropositivity and risk factors at herd and individual level. The herd and individual seroprevalences were 4.1 and 2.0 %, respectively. Herds with a history of abortions were found to be associated with seropositivity [odds ratio (OR) = 5.3; 95 % confidence interval (CI), 1.3–21.3]. Large herds with more than eight cattle were more likely to be seropositive compared to smaller herds with one to two cattle (OR = 13.9; 95 % CI, 1.6–119). The number of calves produced per cow (indicating age) was found to be associated with seropositivity. Younger cows with one to two produced calves were less likely to be seropositive compared to older cows with more than six produced calves (OR = 0.24; 95 % CI, 0.06–1.0). Neither introduction of new cattle to the herd nor communal grazing was associated with seropositivity. This study shows that infection with *Brucella* (1) is present in small-scale urban and peri-urban dairy farming in Tajikistan and (2) has significant negative effects on reproductive performance in this farming system and (3) that some previously known risk factors for seropositivity in rural farming system were absent here.

## Introduction

The bacterial disease brucellosis is a zoonosis affecting public and livestock health worldwide (Pappas et al. 2006). In humans, the infection can cause severe chronic illness in various organs and tissues, with osteoarticular disease being the most common complication (Solera et al. 1999; Pappas et al. 2006). The most prevalent routes of human infection are through consumption of unpasteurized milk products and close contact with infected animals (Young 1995). In livestock, brucellosis mainly causes reproductive disorders such as abortion and male infertility. Large numbers of bacteria are excreted in the birth fluids of an infected animal, and the disease is mainly spread through a direct contact when an infected female aborts or gives birth. Notably, female cattle of breeding age are more susceptible to infection with *Brucella abortus* than younger animals (Nicoletti 1980). There are several different *Brucella* species and *B. abortus* (mainly infecting cattle), *Brucella melitensis* (mainly infecting sheep and goats), and *Brucella suis* (mainly infecting swine) that are considered the most important in livestock. These *Brucella* spp. are highly pathogenic for humans, and cross infections to other animal species can occasionally occur (Quinn et al. 2002).

Brucellosis in livestock has been eradicated from several countries but still remains endemic in many parts of the world. The incidence of human and animal brucellosis is reported to be increasing in many Central Asian countries including Tajikistan, where the incidence of human brucellosis measured in annual cases per million of population was 211.9 in 2006 (Pappas et al. 2006). Uncontrolled movement of livestock, increasing number of small farm units, and insufficient disease control are believed to be the major reasons for this development (Jackson et al. 2007). In Tajikistan, as in many low-income countries, there is a substantial small-scale dairy farming sector in the urban and peri-urban areas of the major cities. This practice is an opportunity for dairy farmers to improve their livelihood (Jackson et al. 2007). However, if the dairy cows are infected with *Brucella*, this opportunity may turn into a severe public health threat. Furthermore, *Brucella* causes major economic losses primarily due to reduction in milk production and increased rate of abortions (Nicoletti 1980). It has been shown that the economic burden of brucellosis is greatest in low-income countries (McDermott et al. 2013).

A nationwide serological survey focusing on brucellosis in sheep and goats was performed in Tajikistan in 2003 (Jackson et al. 2007). The survey included 620 dairy cows from governmental farms as well as small private herds in urban-located villages in the two major cities Dushanbe and Kurgan Tube. The seroprevalences in cattle, sheep, and goats were estimated to be 2.1, 5.8, and 5.5 %, respectively. During the same year, the Food and Agricultural Organisation (FAO) of the UN initiated a brucellosis control program in several regions with high prevalence of brucellosis in Tajikistan. The program included mass vaccination of sheep and goats with Rev 1 *B. melitensis* live attenuated vaccine (FAO 2012; Ward et al. 2012). The region around the capital Dushanbe was not included in the vaccination program, nor were cattle (Ward et al. 2012).

The objectives of the current study were to assess and map the *Brucella* seroprevalence in dairy cows in urban and peri-urban small-scale farming in a low-income country and to identify risk factors associated with seropositivity.

## Materials and methods

### Study area and study population

**Fig. 1:**
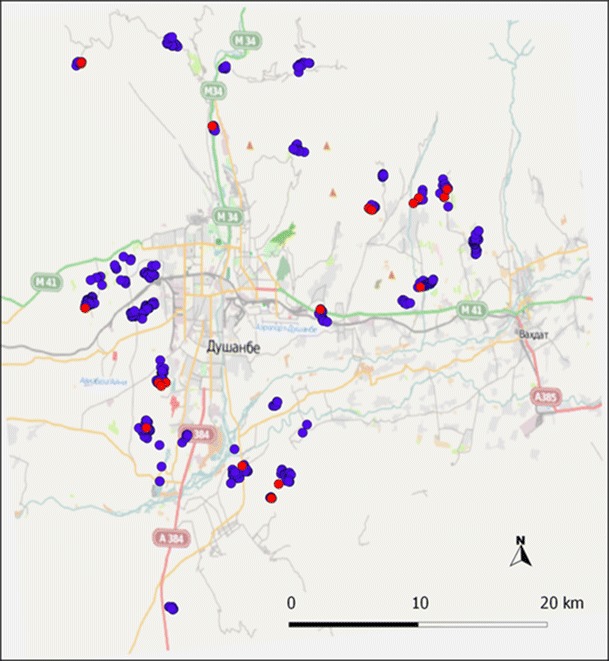
Map over the study area and *Brucella* serology results at herd level (*n* = 441). *Brucella* seropositive herds (*n* = 18) are represented by a *red point* and seronegative herds (*n* = 423) with a *blue point*. © OpenStreetMap contributors (www.openstreetmap.org)

This cross-sectional study was conducted in the urban and peri-urban areas of the capital Dushanbe with a radius of <20 km from the central part of the city (Fig. 1). Approximately 700,000 people live in Dushanbe which is located in the western part of the country surrounded by mountains to the north and a lowland area to the south (UN 2013). According to local legislation, no livestock are permitted in the most central part of the city.

There are approximately 220 villages with 45,000 dairy cows in the study area, which is dominated by small-scale farming, commonly with one to three dairy cows per herd. The average-sized village has about 100 herds with a range from 20 to 200 herds. Some of the villages have access to limited pastures or practice a zero-grazing system, while others have access to vast pastures where communal grazing is common during the grazing season from the end of May to the beginning of October. The majority of the dairy cows in the study population are of local breed. These are small cows with an estimated average annual milk production of 3,000 l. There are also cattle resembling small Holstein-Friesian and mixed breeds.

### Study design

The aim was to include a minimum of 384 herds in this cross-sectional study to estimate the seroprevalence of *Brucella* spp. at a herd level with an expected prevalence of 50 %, a confidence interval (CI) of 95 %, and a desired absolute precision of 5 %. In each herd, the aim was to sample all pregnant heifers in the last trimester and all cows as this allows detection of at least one seropositive individual if the herd size is ≤10 animals, and the expected within-herd seroprevalence is 2 % with a 95 % CI (Cannon and Roe 1982). This sampling strategy also enabled the detection of an individual prevalence of 50 % with a CI of at least 95 % and a precision of at least 5 %. Information on the villages housing dairy cows was received from the local official veterinarians. The villages were numbered and selected randomly. The number of villages planned to be visited was constrained by the numbers of days spent doing field work. Accordingly, the aim was set to visit 30 villages. The sampling needed to be performed, while the animals were not out on pasture in order to secure correct registration of epidemiological data per individual cow. If the cows were let out on pasture prior to the arrival of the study team, the closest nearby village was selected as a replacement to facilitate the sampling. The selection of herds within each village was performed on site and based on if the family was home and willing to participate in the study. In each village, as many herds as possible were sampled.

The samples were collected during 3 weeks in May and October 2011, respectively. These time periods were chosen to avoid the cows being on pasture, which would have made sampling difficult. None of the cattle in the study had been vaccinated against brucellosis according to the information from the local official veterinarians.

### Collection of serum samples

The blood samples were collected from the jugular vein into sterile tubes (without additions) and kept cold during transport to the laboratory at the Tajik Agrarian University in Dushanbe. After centrifugation, serum was removed and inactivated at 56 °C for 30 min before storage at −20 °C until transport to the Swedish University of Agricultural Sciences (SLU, Uppsala, Sweden).

### Ethical statement

All animals in this study were treated according to the ethical standards of Tajik Agrarian University (Dushanbe, Tajikistan), and all animal handlings were approved by the rector of the Tajik Agrarian University. Blood collections were carried out in compliance with EU legislation on research involving animals (EU 2010). The dairy farmers were informed about the purpose and the methods of the study. Oral consent was sought from the farmers before commencement of the study on each farm.

### Collection of epidemiological data

Two written questionnaires were used to collect epidemiological data at herd and individual levels. The family member responsible for the daily management of the cows was interviewed in Tajik, Russian, or Uzbek, depending on the person’s native language. All interviews were conducted by the author N.S. assisted by E.L.

At herd level, data on herd size (total number of dairy cows of breeding age and total number of cattle), presence of sheep or goats in the household, type of pasture, introduction of new cattle, and number of abortions during the last year were collected. At individual level, information on breed, age of the cow, and total number of calves produced per cow was collected. A Global Positioning System receiver was used to collect coordinates (latitude/longitude) of all but two included herds (*n* = 441). The spatial distribution of *Brucella* seropositive herds was investigated using QGIS (Quantum GIS 1.7.3 Wroclaw).

Each blood sample was given a unique code. The same code was written on the questionnaires to link them with the blood samples. No data regarding the identity of the farmers were collected, and there was no identification system for the individual cattle. Therefore, no information could be given to individual farmers on the results from the serology study related to their own cattle. Participation in the study was on a voluntary basis, and the response to the questionnaires constituted the participants’ written consent.

### Serological analyses

Detection of antibodies for *Brucella* was performed with commercial indirect enzyme-linked immunosorbent assay (I-ELISA) (SVANOVA Biotech AB, Uppsala, Sweden) at the SLU in Uppsala, Sweden, according to instructions from the manufacturer. Samples positive in I-ELISA were confirmed with competitive ELISA (C-ELISA) according to recommendations from the World Organisation for Animal Health (OIE 2009). A sample was considered seropositive for *Brucella* when positive on both I-ELISA and C-ELISA. The methods used do not distinguish the different *Brucella* spp.

For I-ELISA, samples were considered positive if percent positivity (PP) is ≥40. The PP was calculated as (Mean OD_sample_/Mean OD_positive control_) × 100 where OD is the optical density.

For C-ELISA, samples were considered positive if percent inhibition (PI) is ≥30. The PI was calculated as 100 − ((Mean OD_samples_ × 100)/(Mean OD_conjugate control_)).

Test validation was performed with positive and negative controls according to instructions from the manufacturer, and all samples were run in duplicates. If a plate validation failed, it was rerun.

### Statistical analyses

The data were entered in Excel software (Microsoft), and statistical analyses were conducted in SAS version 9.2 (Cary, NC, USA). Spearman correlation tests were conducted to identify a possible correlation between the continuous variables. All variables were initially screened in univariable logistic regression. Two multivariable logistic regression models were used to investigate an association between seropositivity and all risk factors at herd and individual level. Manual backward elimination was used until all remaining variables showed a *P* value of ≤0.10. The models were investigated for interactions between all included variables in the final models. Confounding was investigated by adding the eliminated variables in the final models. A variable was considered to be a confounder if it changed the coefficient of the significant variables by >25 %. The fit of the models were assessed using Hosmer and Lemeshow goodness-of-fit test. At individual level, the final model accounted for clustering of animals using generalized estimating equations (GEEs).

## Results

### Description of herds

In total, 904 serum samples were collected from dairy cows of breeding age belonging to 443 herds in 32 villages. One randomly selected village was excluded from the study because all the cows were let out on communal grazing before the arrival of the study team, and the closest nearby village was selected as replacement. Only two farmers refused to participate in the study, and three farmers were not at home and hence unable to participate. The most remote herd was located approximately 18 km from the center of Dushanbe. The median herd size was two (1–15) cows and four (1–24) cattle in total. Twenty percent of the herds possessed small ruminants, and only 10 % reported having bought new cattle during the last year. A slight majority of included cows were kept on limited pastures or tethered on a pasture (51 %). Few herds (2 %) practiced a zero-grazing system. Four percent of the herds claimed having cows that aborted during the last year (Table 1). Most cows were of local breed (Table 2), the median age was 5 years (2–22), and the median number of calves produced per cow was 3 (1–20).

**Table 1:** Descriptive results and the relationship between potential risk factors and *Brucella* seropositivity at herd level (*n* = 443) using univariable logistic regression analyses

Variable	Category	Number (%)	Seropositive number (%)	*P* value
Number of cows				0.05^a^
	1 and 2	326 (74)	9 (3)	Reference
	3 and 4	81 (18)	5 (6)	0.9
	≥5	36 (8)	4 (11)	0.08
Number of cattle				0.06^a^
	1 and 2	121 (27)	1 (1)	Reference
	3 and 4	167 (38)	7 (4)	0.7
	5–7	100 (23)	4 (4)	0.8
	≥8	55 (12)	6 (11)	0.007
Sheep/goats	Yes	90 (20)	5 (6)	0.4^a^
	No	353 (80)	13 (4)	
Pasture type				0.1^a^
	Communal grazing	207 (47)	12 (6)	
	Limited pasture/tethered	226 (51)	5 (2)	
	Zero grazing	10 (2)	1 (10)	
Purchase new cattle	Yes	45 (10)	2 (4)	0.9^a^
	No	398 (90)	16 (4)	
Abortion	Yes	17 (4)	3 (18)	0.01^a^
	No	426 (96)	15 (4)	

^a^Likelihood ratio test

**Table 2:** Descriptive results and the relationship between potential risk factors and *Brucella* seropositivity at individual level using univariable logistic regression analyses

Variable	Category	Number (%)	Seropositive number (%)	*P* value
Breed (*n* = 904)	Local	823 (91)	16 (2)	0.7
	Other^a^	81 (9)	2 (2)	
Age of the cow in years (*n* = 743)	≤4	202 (27)	4 (2)	0.4
	>4–6	308 (41)	4 (1)	0.2
	>6–8	154 (21)	4 (3)	0.6
	>8	79 (11)	3 (4)	Reference
Number of calf/calves produced per cow (*n* = 787)	≤2	357 (45)	5 (1)	0.051
	>2–4	277 (35)	4 (1)	0.07
	>4–6	100 (13)	3 (3)	0.4
	>6	53 (7)	3 (6)	Reference

^a^Other breed includes mixed breed and cattle resembling Holstein-Friesian

### Seropositivity to *Brucella* spp.

Eighteen of 443 herds had at least one seropositive cow, resulting in a herd seroprevalence of 4.1 % (95 % CI, 2.6–6.3 %). At individual level, the seroprevalence was 2.0 % (*n* = 18, 95 % CI, 1.3–3.1 %). Additional four individual samples were positive on I-ELISA, but negative on C-ELISA and were therefore considered to be negative for brucellosis.

The distribution of *Brucella*-seropositive herds is shown in Fig. 1.

### Risk factors associated with *Brucella* seropositivity

In two of the variables at herd level, the total number of dairy cows of breeding age and the total number of cattle were correlated (coefficient 0.8, *P* ≤ 0.001), and only the latter was included in the logistic model, as it was believed that this variable contained more relevant information regarding the transmission of infection. At individual level, the age of the cow and the number of calves produced per cow were correlated (coefficient 0.9, *P* ≤ 0.001). The variable age of the cow was excluded due to more missing data compared to the number of calves per cow. The results from the univariable logistic regression analyses at herd and individual level are shown in Tables 1 and 2. The result from the multivariable logistic regression at herd level shows that abortions were significantly associated with seropositivity (*P* = 0.02), and herd size was borderline significant (*P* = 0.07). Herds with more than eight cattle were significantly associated with seropositivity (*P* = 0.02) compared with the herd size of one to two cattle (Table 3). The *P* value was 0.9 for Hosmer and Lemeshow goodness-of-fit test, which indicates a good fit of the model. At individual level, there were two categories within the variable number of calves produced per cow that were borderline significant. These were the categories with one to two calves produced per cow (*P* = 0.051) and three to four calves produced per cow (*P* = 0.07) compared to the category with more than six calves produced per cow (Table 4). No interactions or confounding was found in any of the models.

**Table 3:** Relationship between potential risk factors and *Brucella* seropositivity at herd level (*n* = 443) using multivariable logistic regression analyses

Variable	Category	*β*	SE	*P*	OR (95 % CI)
Abortion	Yes	1.7	0.7	0.02^a^	5.3 (1.3–21.3)
	No				
Herd size cattle				0.07^a^	
	1 and 2				Reference
	3 and 4	1.6	1.1	0.14	5.0 (0.6–41.5)
	5–7	1.6	1.1	0.17	4.8 (0.5–43.9)
	≥8	2.6	1.1	0.02	13.9 (1.6–119)

^a^Likelihood ratio test

**Table 4:** Relationship between potential risk factors and *Brucella* seropositivity at individual level (*n* = 904) using multivariable logistic regression analyses

Variable	Category	*β*	SE	*P*	OR (95 % CI)
Nr of calves	≤2	−1.4	0.7	0.051	0.24 (0.06–1.0)
	>2–4	−1.4	0.8	0.067	0.25 (0.06–1.1)
	>4–6	−0.6	0.8	0.44	0.53 (0.11–2.6)
	>6				Reference

## Discussion

*Brucella* infection, defined by seropositivity, is present in small-scale dairy farming in an urban and peri-urban area of Tajikistan, and seropositivity was positively associated with abortions, herd size, and the number of calves produced per cow.

The herd and individual seroprevalences were 4.1 and 2.0 %, respectively. Because none of the cattle in this study were vaccinated against brucellosis, seropositivity was considered to be caused by natural exposure. Results on the individual seroprevalence support those from a previous study comprising small- as well as large-scale herds in Tajikistan about a decade ago (Jackson et al. 2007). A nationwide study conducted in the neighboring country Kyrgyzstan showed results similar to this study with an individual seroprevalence in cattle of 2.8 % (Bonfoh et al. 2012). However, higher seroprevalences have been shown among cattle in rural areas in other parts of the world such as Jordan with an individual seroprevalence of 6.5 % (Al-Majali et al. 2009), Sri Lanka with an individual seroprevalence of 4.7 % (Silva et al. 2000), and Zambia with an individual seroprevalence ranging from 14 to 28 % (Muma et al. 2006). Notably, few seroprevalence studies conducted on brucellosis in cattle have focused on urban or peri-urban farms. These farms are often located close to large markets, and contaminated products may thus be spread effectively to large groups of consumers. For instance, trading of raw milk and raw milk products is a common practice among the farmers living close to the capital in Tajikistan (Lindahl et al., unpublished data).

In this study, it was found that herds with a history of abortions were associated with *Brucella* seropositivity. This finding is in correspondence with the biology of *Brucella*, and similar results have been described in other field studies (McDermott et al. 1987; Silva et al. 2000; Al-Majali et al. 2009; Matope et al. 2011) and reconfirm that brucellosis in dairy cattle is not only a public health issue. It is generally acknowledged that abortions and decreasing milk yield can be of major economic importance in an infected herd (Corbel 1988).

Large herds with eight or more cattle were more likely to be seropositive compared to herds with one or two cattle. This is consistent with other studies from Kenya, sub-Saharan Africa, and Jordan (Kadohira et al. 1997; McDermott and Arimi 2002; Al-Majali et al. 2009). It is likely that the large herds provide more opportunities for transmission through contact with aborted materials from infected cows.

At individual level, the number of calves produced per cow was found to be associated with seropositivity. Younger cows with one or two produced calves were less likely to be seropositive compared to older cows with more than six produced calves. Susceptibility to *Brucella* is commonly more associated with sexual maturity than age (Nicoletti 1980). Other studies have found that cows older than 3 years (Silva et al. 2000) and 4 years (Al-Majali et al. 2009) were more likely to be seropositive compared to younger animals. In this study, where we only sampled sexually mature dairy cows, the older cows had lived a longer time at risk of being exposed to infection compared to the younger sexually mature cows.

Interestingly, there was no significant difference in seroprevalence between different types of pasture in this study, although other studies have found that communal grazing might be a risk factor for the transmission of the disease (Salman and Meyer 1984). The finding in the current study might be attributable to the fact that most farmers keep their cows within the farms instead of on the pasture 1 month prior to and during calving and, hence, might decrease the risk of contaminating the pasture with *Brucella* bacteria shed in birth fluids and placentas. Likewise, this study did not show any evidence of association between introduction of new cattle into a herd and seropositivity for *Brucella*. One explanation for this might be the small number of herds that had purchased new cattle. This practice is otherwise known to increase the risk of transmission of infection between herds (Corbel 1988).

A nationwide study from Armenia showed a widespread but uneven distribution of *Brucella* seropositivity in cattle (Porphyre et al. 2010). A limitation for investigating spatial patterns in the current study was that it only focused on the area close to the capital city in Tajikistan. An extended study covering more variation in farming practices and agroecology would probably be required to demonstrate spatial patterns of *Brucella* seropositivity. Potential biases in the sampling might arise due to the exclusion of certain villages and herds. These exclusions were caused either by the cows being let out on pasture or the farmers not being at home. As described in the “Results” section, only one village was excluded and, additionally, three herds. The results are, therefore, considered to give a representative picture of the presence of factors associated with brucellosis among the population of urban and peri-urban dairy herds in the study area.

Urban and peri-urban dairy farming offers an important opportunity to improve the livelihood of many people in low-income countries. However, brucellosis among livestock can turn this practice into a major public health threat. Therefore, there is a need for studies describing the prevalence of brucellosis and risk factors for transmission as well as information about economic losses caused by brucellosis in order to encourage control measures for this neglected zoonosis.
